# Characterization of Tauopathies and Aggregation Patterns in Brain Regions

**DOI:** 10.1002/alz70861_108969

**Published:** 2025-12-23

**Authors:** Adam Ping Liu

**Affiliations:** ^1^ University of California Los Angeles, Los Angeles, CA USA

## Abstract

**Background:**

Clinical differences in Parkinson’s Disease (PD) are highly impacted by distinct patterns of tau aggregation in various neuropathologies. Previous studies have shown pathological tau aggregation to be a defining feature of tauopathies, but their accumulation and the inherent vulnerability of select brain regions present challenges to understanding PD progression. These aggregation patterns, known as tau strains, possess a myriad of conformations that may affect tau transmission and neurodegeneration, though these effects are not clearly understood.

**Method:**

To investigate this, we developed advanced mouse models to identify tau strains and analyze their varying degrees of pathology. We will use bioimaging analysis software to illustrate different areas of the brain and annotate their local pathologies following the injection of pathological tau into diverse mice brain regions. By qualitatively observing these images and quantitatively classifying their neuropathological characteristics, we will be able to examine certain tau aggregates that will help us in predicting the progression of strain‐specific tauopathies.

**Result:**

Morphology of tau strains varied greatly depending on strain, dictating disease phenotype. However, pathological tau was consistently characterized by aggregation in neurofibrillary tangles and hyperphosphorylation. PD‐tau was primarily composed of the 3R and 4R isoforms.

**Conclusion:**

Different tau strains induced various distributions of aggregation, demonstrating the profound diversity of tauopathies. Understanding the mechanisms of this pathology could be essential to treating PD and other tauopathies in the future.

